# CanPrevent: a telephone-delivered intervention to reduce multiple behavioural risk factors for colorectal cancer

**DOI:** 10.1186/1471-2407-12-560

**Published:** 2012-11-27

**Authors:** Anna L Hawkes, Tania A Patrao, Anita Green, Joanne F Aitken

**Affiliations:** 1 School of Public Health and Social Work, Queensland University of Technology, Victoria Park Road, Kelvin Grove, Brisbane, Queensland, 4059, Australia; 2 Viertel Centre for Research in Cancer Control, Cancer Council Queensland, PO Box 201, Spring Hill, Brisbane, Queensland, 4004, Australia; 3 The University of Queensland Health Service, The University of Queensland, Brisbane, Australia

**Keywords:** Colorectal cancer, Multiple health behaviour change intervention, Lifestyle, Physical activity, Telephone, Prevention, Family history

## Abstract

**Background:**

This pilot study aimed to test the acceptability and short-term effectiveness of a telephone-delivered multiple health behaviour change intervention for relatives of colorectal cancer survivors.

**Methods:**

A community-based sample of 22 first-degree relatives of colorectal cancer survivors were recruited via a media release. Data were collected at baseline and at six weeks (post-intervention). Outcome measures included health behaviours (physical activity, television viewing, diet, alcohol, body mass index, waist circumference and smoking), health-related quality of life (Short Form-36) and perceived colorectal cancer risk. Intervention satisfaction levels were also measured. The intervention included six telephone health coaching sessions, a participant handbook and a pedometer. It focused on behavioural risk factors for colorectal cancer [physical activity, diet (red and processed meat consumption, fruit and vegetable intake), alcohol, weight management and smoking], and colorectal cancer risk.

**Results:**

From baseline to six weeks, improvements were observed for minutes moderate-vigorous physical activity (150.7 minutes), processed meat intake (−1.2 serves/week), vegetable intake (1 serve/day), alcohol intake (−0.4 standard drinks/day), body mass index (−1.4 kg/m2), and waist circumference (−5.1 cm). Improvements were also observed for physical (3.3) and mental (4.4) health-related quality of life. Further, compared with baseline, participants were more likely to meet Australian recommendations post-intervention for: moderate-vigorous physical activity (27.3 vs 59.1%); fruit intake (68.2 vs 81.8%); vegetable intake (4.6 vs 18.2%); alcohol consumption (59.1 vs 72.7%); body mass index (31.8 vs 45.5%) and waist circumference (18.2 vs 27.3%). At six weeks participants were more likely to believe a diagnosis of CRC was related to family history, and there was a decrease in their perceived risk of developing CRC in their lifetime following participation in CanPrevent. The intervention retention rate was 100%, participants reported that it was highly acceptable and they would recommend it to others at risk of colorectal cancer.

**Conclusions:**

Positive behaviour change achieved through this intervention approach has the potential to impact on the progression of CRC and other cancers or chronic diseases. A large scale randomised controlled trial is required to confirm the positive results of this acceptability and short-term effectiveness study.

**Trial registration:**

ACTRN12612000516886

## Background

Colorectal cancer (CRC) is one of the leading causes of cancer morbidity and mortality in the industrialized world [1]. Most cases (93%) occur in persons aged 50 years or more [2] and it often co-exists with other chronic diseases related to health behaviours including obesity, type 2 diabetes mellitus and cardiovascular disease [3,4]. Behavioural risk factors including physical inactivity [5], diet [6-8] and obesity [9-11] play a pivotal role in the aetiology of CRC, and it has been estimated that at least 70% of CRC may be prevented with moderate behavioural changes [12]. In particular, reductions in the consumption of alcohol and red and processed meat, weight loss and increased levels of physical activity may translate into significant reductions in the incidence of CRC [12]. Importantly, these lifestyle changes also decrease risk of other cancers as well as type 2 diabetes mellitus and cardiovascular disease [13,14], therefore behavioural improvements result in overall health benefits.

Individuals with a family history of CRC have a significantly elevated risk of developing CRC [15]. Epidemiological studies indicate that first degree relatives of CRC survivors (parents, siblings, or offspring) have a 1.6 to 8-times higher life time risk of CRC than those without a family history, the strength of the relationship varying according to age at diagnosis in the index case, type of relative, and the number of relatives affected [15]. Furthermore, a combination of familial predisposition and unhealthy behaviours increases risk of CRC considerably [16].

Despite the evidence, research has shown that most people are unaware of the association between behavioural risk factors and CRC risk [17], and individuals identified at high risk of CRC do not generally make voluntary behavioural changes [18]. One study found that first degree relatives of CRC survivors attributed their risk of CRC to physiology (27%) or family history (25%), whilst only 16% believed behavioural risk factors were of importance [19]. As such, it is important to educate people about the importance of their health behaviours and to support those at high risk of CRC to make improvements to reduce their risk of the disease. To our knowledge, there are no programs routinely available to support individuals considered at increased risk of CRC. The absence of specific programs for this population group remains a missed opportunity as national policy supports cancer reduction and the evidence suggests that behaviour change programs targeting high risk groups may be more effective than those targeting the population at large [20,21].

There is also a paucity of research investigating educational or supportive interventions specifically for those at high risk of CRC with, to our knowledge, just two published studies in the field specifically targeting those with colorectal adenomas [20,21]. *Bowel health to better health* was a trial of a three month minimal contact intervention (one face to face session followed by three personalised mailings; n=74). The intervention included lifestyle advice, goal setting and social support to promote increases in physical activity, fibre, fruit and vegetable intake. However, the study was limited by a low response rate (51%), and intervention effects were observed for fibre intake alone [20]. Project PREVENT was a trial of a tele-based counselling intervention based on Social Cognitive Theory [22] to improve multiple risk factors (red meat, fruit, vegetable, multivitamin and alcohol intake, smoking, and physical activity; n=1247). Intervention effects were observed for multiple risk factors (including multivitamin and red meat intake), and intervention participants tended to have a lower rate of regression in their levels of physical activity than usual care participants. However, there were no direct intervention effects on smoking, alcohol, fruit or vegetable intake, and the study was limited by the inclusion of participants who were highly educated [21].

Behavioural risk factors for CRC are interrelated in terms of the psychological, social and environmental factors that reinforce them [23] (for example, those who eat high-fat diets are more likely to be sedentary and to be cigarette smokers) [22,24,25]. Also, previous investigations [24,26] have shown that change in one behavioural risk factor may serve as a stimulus or gateway for change in other health behaviours. However, few CRC studies have intervened on multiple behaviours simultaneously. It represents a challenge from an intervention perspective, but provides an important opportunity to maximise the potency of cancer prevention interventions as the complex and multifactorial process of carcinogenesis suggests that several behavioural changes may be needed to significantly reduce risk. Thus multiple risk factor interventions warrant further study [21].

Theory-based behavioural interventions have been shown to be most effective and Social Cognitive Theory is widely used [22,27]. In contrast, the CanPrevent intervention used specific strategies from Acceptance Commitment Therapy (ACT), which is an empirically based third generation cognitive behavioural approach that uses acceptance and mindfulness strategies, and commitment and behaviour change strategies to produce psychological flexibility: the ability to defuse from difficult thoughts and accept difficult feelings while persisting in values-based action [28-31]. This provided an alternative to existing intervention approaches by overcoming internal barriers to making lifestyle improvements by emphasizing the role of emotions and thoughts in the maintenance of good self-management of lifestyle factors [32]. To date, ACT interventions have been successfully used to enhance quality of life and promote positive lifestyle behaviours for a range of health conditions (chronic pain [33], diabetes [34], epilepsy [35], smoking [36], and obesity or weight management [37-39]) but this approach has not previously been used for those at high risk of CRC.

Previous researchers have investigated a range of delivery modes for behavioural interventions (face to face, telephone, internet and paper-based delivery) and telephone-delivered interventions have been shown to be highly acceptable [40], improve behavioural outcomes in the short term [41,42] for cancer survivors, and there is a solid evidence base supporting the efficacy of telephone based interventions for physical activity and dietary behaviour change [43,44]. Importantly, in Australia, approximately 96% of the population live in a household with at least one telephone connection, hence this approach appeared viable for the current study [45].

This is the first pilot study of the acceptability and short-term effectiveness of a novel theory-based telephone-delivered multiple risk factor intervention to improve behavioural risk factors, health-related quality of life (HRQoL) and perceived risk of CRC for first degree relatives of CRC survivors.

## Methods

### Participants

From February to March 2011 (eight weeks) adults resident in Queensland, Australia who were a first degree relative of someone with a confirmed diagnosis of primary CRC (C18-C20, C218) were sourced through Queensland based media advertisements (print, radio, internet). Eligibility criteria included: (i) ability to understand and provide written informed consent in English; (ii) no current or previous diagnosis of CRC; (iii) no medical conditions that would limit adherence to an unsupervised lifestyle program; (iv) a telephone; and (v) those who had at least one poor health behaviour consistent with Australian recommendations [46-49] [do not achieve ≥150 minutes moderate-vigorous physical activity per week; *or* do not adhere to a healthy diet (indicated by >4 serves red meat/week, *or* <2 serves fruit per day, *or* <5 serves vegetables per day); *or* consume >*2* standard drinks per day; *or* are overweight (body mass index or BMI ≥25). Participants were screened for eligibility prior to recruitment.

### Data collection

Data were collected at baseline (pre-intervention) and at six weeks (post-intervention) by dedicated computer assisted telephone interviewers using measures that have previously been used in longitudinal studies over the phone [43,50-52]. Information was collected on outcomes targeted by the intervention including: behavioural risk factors [physical activity, television (TV) viewing, diet, alcohol, BMI, waist circumference and smoking], generic HRQoL and perceived CRC risk. At the completion of the intervention, participants were mailed a self-reported survey to assess satisfaction with the intervention.

#### Physical activity

We used a modified version of the leisure score index of the Godin Leisure-Time Exercise Questionnaire that has been shown to be a reliable and valid self-report measure of physical activity. The leisure score index contains three questions that assess the average frequency of mild, moderate, and strenuous exercise during free time in a typical week. The modified version of the leisure score index also provides average duration of physical activity [53]. Participants were categorised in to ≥ 150 minutes/week moderate to vigorous physical activity or <150 minutes/week moderate-vigorous physical activity consistent with Australian recommendations [46].

#### TV viewing

Within epidemiological and health behaviour research, measurement of adults’ sedentary behaviour has often focused on TV viewing, one of the most frequently reported leisure-time activities [54]. Participants provided an estimate of the total time spent watching TV, on an average day, over the past month. Self-reported TV viewing has been shown to be a reasonably reliable and valid measure for adults [55].

#### Diet and alcohol

We used brief questions about diet and alcohol behaviour (red meat, processed meat, vegetable, fruit, alcohol intake) based on the Cancer Council New South Wales validated and commonly used items for assessing diet and alcohol in cancer patients [56]. Consistent with national recommendations [57] participants were categorised in to: (i) red meat intake ≤4 serves/week or >4 serves/week; (ii) vegetable intake ≥5 serves/week or <5 serves/week; (iii) fruit intake ≥2 serves/week or <2 serves/week; and (iv) alcohol consumption ≤2 standard drinks/week or >2 standard drinks/week. There are no specific national recommendations for processed meat intake (‘*avoid processed meats*’) [57].

#### Smoking

We used brief validated questions about smoking behaviour developed by the Cancer Council New South Wales for assessing smoking behaviour in cancer patients [56].

#### BMI

Self-reported height and weight were recorded and they were categorised as healthy weight (BMI 18.5 – 24.9 kg/m2), overweight (BMI 25.0 – 29.9 kg/m2), or obese (BMI ≥ 30.0kg/m2) consistent with national recommendations [58]. Participants were given an instruction sheet on how to accurately take measurements and they provided self-reported height and weight which has been shown to provide accurate results in previous telephone-delivered intervention trials [43,44,50].

#### Waist circumference

Participants were provided with tape measures and an instruction sheet on self measurement of waist circumference (cm) and prior to the telephone interview, as previous investigators have demonstrated a high correlation between self-reported and technician-recorded waist circumference in males and females [59].

Participants were classified as low risk (≤94cm men, ≤80cm women), increased risk (>94cm men, >80cm women) or greatly increased risk (>102cm men, >88cm women) consistent with national recommendations [60].

#### HRQoL

We used the Short Form-36 (SF-36), a widely used measure that has published norms for the Australian general population [61,62]. The SF-36 provides a physical- and mental- HRQoL summary measure suitable to measure the impact of the intervention on patients’ wellbeing. It also provides eight sub-scales including: physical functioning, role-physical, bodily pain, general health, vitality, social functioning, role-emotional and mental health [61,62].

#### CRC screening and perceived risk of CRC

Participants were asked whether they had ever been screened for CRC and to provide the approximate date and screening test used, they were also asked whether they intended to be screened in the future. In addition, participants were asked about their perceived risk of CRC using a modified version of brief validated questions [19] [‘*What proportion of people in the general population do you believe are diagnosed with CRC due to family history*?’, ‘*What do you think your chances are of ever developing CRC in your lifetime*?’ (scored 0-100%)].

#### Intervention satisfaction

Participants were asked about their satisfaction (scored on a four point likert scale from ‘*highly unsatisfied*’, ‘*unsatisfied*’, ‘*satisfied*’ to ‘*highly satisfied*’) with the CanPrevent program overall, the health coaches and the CanPrevent Handbook. Participants were also asked to indicate (yes, no) whether they would recommend the intervention to others at risk of CRC.

### Intervention

The intervention included six evidence-based telephone health coaching sessions delivered by study-trained health professionals (‘health coaches’), and a participant handbook, worksheets and a Yamax SW700 Multifunction Digi-Walker pedometer. Health coaches had tertiary qualifications in nursing, psychology or health promotion. They received six weeks of study-specific training in ACT, behavioural models of health and illness and behaviour change, and Australian recommendations for health behaviours. The program focused on supporting participants to make positive lifestyle changes (physical activity, diet, weight management, alcohol and smoking) and to uptake CRC screening consistent with national guidelines [46-49,57,63,64]. The intervention included an evidence-based approach with strategies drawn from the core components of ACT [28,31]. ACT is an empirically based third generation cognitive behavioural approach that uses acceptance and mindfulness strategies, and commitment and behaviour change strategies to produce psychological flexibility: the ability to defuse from difficult thoughts and accept difficult feelings while persisting in values-based action. Psychological flexibility was established through six core ACT processes: acceptance, cognitive defusion (changing our relationship with thoughts), being present, self-as-context, values and committed action [31]. The approach explicitly taught strategies designed to increase tolerance in the service of goal-directed behaviour, such as healthy eating and physical activity. Importantly, we were not trialling a psychotherapeutic intervention; rather ACT strategies were used to enhance positive lifestyle behaviours.

Intervention strategies included motivational interviewing, problem solving, action planning and goal setting, as well as reviewing and ongoing monitoring to enhance lifestyle change. The health coaching sessions ran over six weeks for one hour each (an introductory session followed by four weekly and one fortnightly session). Intervention sessions included: (i) An introduction session covering: - motivation, expectations, and understanding of CanPrevent; family history and personal circumstances; the role of the health coach; using the CanPrevent Handbook; using the pedometer; and participants were asked to complete a worksheet called “My Healthiest Life Wish List” detailing their health-related values, and to commence tracking their lifestyle factors (physical activity, diet, alcohol, BMI, smoking); (ii) Sessions one to four covered: values and mindfulness, and action planning and goal setting to improve lifestyle factors; (iii) Session five covered: reviewing the previous sessions and action planning and goal setting beyond CanPrevent. Telephone sessions were at no cost to the study participant.

The participant handbook included educational information on health behaviours and the core components of ACT, as well as tracking and monitoring tables for health behaviour change. Using the pedometer, participants were encouraged to achieve 10,000 steps/day as the recommended goal [46,65], and to track and monitor their steps throughout the intervention.

The study protocol was approved by the University of Queensland Behavioural Social Sciences Ethics Review Committee. To ensure fidelity of intervention delivery, the intervention protocol was detailed in a manual, all sessions were scripted and all intervention calls were audio-taped and reviewed against a session checklist based on the objectives for each session. The health coaches also met with the lead investigator with expertise in behaviour change for bi-weekly supervision sessions.

### Statistical analyses

Data from the telephone interviews were checked for out-of-range or inconsistent data. Descriptive statistics [n (%) and mean (standard deviation or SD)] were used to describe variables. T-tests were used to measure the change from baseline to six weeks in behavioural variables and HRQoL. Mean differences (95% confidence interval or CI) with corresponding p-values have been presented. From baseline to six weeks, Chi^2^ tests were used to compare the proportion of participants meeting the national recommendations for behavioural variables and to compare perceived risk of CRC, corresponding p values are presented. Statistical significance was set at p≤0.05. All analyses were conducted using Stata statistical software (Statacorp, College Station, TX, USA).

## Results

The first 28 potential participants were screened for eligibility and 22 eligible participants (79%) were recruited to the study. We continued to receive expressions of interest from potential participants (total n=61) over the study recruitment period, however sample size was not increased as we had reached our required sample size for a small pilot study to provide useful information about the acceptability and short-term effectiveness of the intervention. Non-participants were sent a covering letter and standard Cancer Council Queensland resources on reducing risk of cancer. Reasons for ineligibility included: previous diagnosis of CRC (n=2), not a first degree relative of a CRC survivor (n=2), or meeting the Australian recommendations for health behaviours (n=2). All 22 participants received 6 health coaching sessions over the 6 week intervention period. Baseline demographic variables are presented in Table 1. In brief, participants were middle aged (mean age=47.3yrs), and the majority (82%) were female, born in Australia (91%), married or in a de-facto relationship (73%) and had completed high school (96%). All participants had at least 1 first degree relative diagnosed with CRC with 4 participants (18%) having more than 1 first degree relative with CRC.

**Table 1:** Demographic variables

**Variable**	**n=22**
Age, mean (SD)	47.3 (13.4)
Gender, n (%) female	18 (81.8)
Born in Australia, n (%)	20 (90.9)
Aboriginal/Torres Strait Islander, n (%)	0
English speaking, n (%)	22 (100)
Married/De-facto, n (%)	16 (72.7)
Completed at least high school, n (%)	21 (95.5)
Employed, n (%)	13 (59.1)
Private Health Insurance, n (%)	19 (86.4)
Household income, n (%)	
– < $25,000	4 (18.2)
– $25,000-$40,000	1 (4.6)
– $40,000-$65,000	6 (27.3)
– $65,000-$100,000	5 (22.7)
– >$100,000	1 (4.6)
Number of First Degree Relatives >1, n (%)	4 (21.1)

### Health behaviours

Mean change in health behaviours from baseline to 6 weeks for minutes moderate-vigorous physical activity, TV viewing, diet, alcohol intake, BMI and waist circumference are shown in Table 2. We observed an improvement in: moderate-vigorous physical activity (150.7 minutes); TV viewing (−1.4 hours/week); processed meat intake (−1.2 serves/week), fruit (0.3 serves/day) and vegetable intake (1.0 serve/day), BMI (−1.4 kg/m^2^) and waist circumference (−5.1 cm). At baseline, 7 participants were former smokers and 1 participant was a current smoker. The median number of cigarettes smoked by the former and current smokers was 14 (range 3–30) with the median starting age of 18 years (range 10–25). During the intervention period, the 1 current smoker quit smoking. Further, from baseline to 6 weeks, participants were more likely to meet the Australian recommendations for moderate-vigorous physical activity (27.3 to 59.1%), red meat (86.4 to 90.9%), fruit (68.2 to 81.8%),vegetables (4.6 to 18.2%), alcohol (59.1 to 72.7%), BMI (31.8 to 45.5%) and waist circumference (18.2 to 27.3%; Table 3).

**Table 2:** **Mean change in moderate to vigorous physical activity** (**MVPA**), **TV viewing**, **diet** (**red meat**, **processed meat**, **fruit**, **vegetables**), **alcohol**, **body mass index and waist circumference from baseline to six weeks**

**Variable**	**Baseline mean(SD)**	**Six weeks mean(SD)**	**Six weeks – baseline (95%CI)**	***p***
MVPA, mins/week	66.6 (21.1)	217.3 (34.7)	150.7 (22.7 110.5)	<0.01
TV viewing, hrs/week	12.9 (1.7)	11.5 (1.6)	-1.4 (-4.0 1.2)	0.28
Diet				
Red meat, serves/week	2.8 (1.5)	2.8(1.5)	0.02 (-0.6 0.6)	0.93
Processed meat, serves/week	2.0 (1.8)	0.9(1.2)	-1.2 (-1.8 -0.5)	<0.01
Fruit, serves/day	1.8 (0.9)	2.2 (1.2)	0.3 (-0.3 0.9)	0.27
Vegetables, serves/day	2.5 (1.3)	3.5 (1.1)	1.0 (0.4 1.6)	<0.01
Alcohol, standard drinks/day	1.9 (0.3)	1.5(0.3)	-0.4 (-0.8 0.1)	0.13
Body mass index, kg/m^2^	30.4 (7.4)	29.0 (6.1)	-1.4 (-2.3 -0.5)	<0.01
Waist circumference, cm	97.5 (18.4)	92.4 (15.7)	-5.1 (-8.3 -2.0)	<0.01

**Table 3:** Proportion of participants meeting the national recommendations for health behaviours at baseline and follow up

**Variable**	**Baseline n (%)**	**Six weeks n (%)**	***p***
MVPA^1^			
≥ 150 mins/week	6(27.3)	13(59.1)	
< 150 mins/week	16(72.7)	9(40.9)	0.02
Red Meat			
≤ 4 serves/week	19(86.4)	20(90.9)	
> 4 serves/week	3(13.6)	2(9.1)	0.12
Fruit			
≥ 2 serves/day	15(68.2)	18(81.8)	
< 2 serves/day	7(31.8)	4(18.2)	0.04
Vegetables			
≥ 5 serves/day	1(4.6)	4(18.2)	
< 5 serves/day	21(95.5)	18(81.8)	0.03
Alcohol			
≤ 2 standard drinks/day	13(59.1)	16(72.7)	
> 2 standard drinks/day	9(40.9)	6(27.3)	<0.01
Body mass index			
Normal weight^2^	7(31.8)	10(45.5)	
Overweight/obese^3^	15(68.2)	12(54.5)	<0.01
Waist circumference			
Low risk^4^	4(18.2)	6(27.3)	
Increased risk^5^	18(81.8)	16(72.7)	<0.01

Moderate to vigorous physical activity. 2. Normal weight (18.5 – 24.9 kg/m2). 3. Overweight/obese (≥25 kg/m2). 4. Low risk (≤94cm men, ≤80cm women). 5. Increased risk (>94cm men, >80cm women).

### HRQoL

From baseline to 6 weeks there was an improvement in physical (3.3) and mental HRQoL scores (4.4). We also observed improvements in physical functioning (2.2), bodily pain (5.8), general health (3.7), vitality (4.8); and role-physical (3.1), social functioning (4.3), role-emotional (3.7) and mental health (4.8; Table 4).

**Table 4:** **Mean change in health**-**related quality of life** (**HRQoL**) **from baseline to six weeks**

**Short form-36 subscale**	**Baseline mean (SD)**	**Six weeks mean (SD)**	**Six weeks - baseline (95%CI)**	***p***
Physical Functioning	50.6 (6.5)	52.8 (4.4)	2.2 (0.9 3.5)	<0.01
Role Physical	47.2 (8.5)	50.2 (7.7)	3.1 (-0.9 7.0)	0.12
Bodily Pain	45.0 (11.4)	50.7 (9.7)	5.8 (1.4 10.1)	0.01
General Health	48.4 (29.6)	52.1 (8.6)	3.7 (0.3 7.1)	0.03
Vitality	47.3 (42.7)	52.0 (49.3)	4.8 (1.4 8.2)	0.01
Social Functioning	48.0 (43.2)	52.3 (49.8)	4.3 (-0.4 9.0)	0.07
Role Emotional	47.0 (14.2)	50.7 (6.5)	3.7 (-2.0 9.4)	0.19
Mental Health	45.8 (13.7)	50.6 (6.8)	4.8 (-0.7 10.3)	0.08
Physical HRQoL	48.6 (1.6)	51.9 (6.6)	3.3 (03 6.3)	0.03
Mental HRQoL	46.4 (14.9)	50.8 (6.9)	4.4 (-1.9 10.7)	0.16

### CRC screening and perceived risk of CRC

At baseline 14 participants had been screened for CRC, while an additional 2 participants were screened during the intervention period. From baseline to 6 weeks [baseline % (SD) vs 6 weeks % (SD), *p* value], we observed an increase in the proportion of participants who believed that a diagnosis of CRC was related to family history [27 (23.9) vs 31.8 (21.6), *p*=0.52], and their perceived risk of developing CRC in their lifetime decreased [49.3 (27.0) vs 39.4 (23.3), *p*=0.20].

### Program satisfaction

100% of participants were highly satisfied with the intervention overall, 74% were highly satisfied with the health coaches, 89% were highly satisfied with the CanPrevent handbook, and 100% stated that they would recommend the intervention to others at risk of CRC.

## Discussion

This report describes the acceptability and short-term effectiveness of CanPrevent, a telephone-delivered theory-based intervention to improve health behaviours for first degree relatives of CRC survivors. We received an overwhelmingly positive response from potential participants with a 100% intervention retention rate. Participants also reported that the intervention was highly acceptable and that they would all recommend it to others at risk of CRC. From baseline to six weeks, we observed improvements in moderate-vigorous physical activity, TV viewing, processed meat intake, fruit and vegetable intake, BMI and waist circumference. and the only current smoker at baseline quit during the intervention. Further, participants were more likely to meet the national recommendations for moderate-vigorous physical activity, fruit, vegetable and alcohol intake, BMI and waist circumference. Participants reported an improvement in the SF-36 summary scores (physical and mental HRQoL), as well as for the SF-36 subscales (role-physical, bodily pain, general health, vitality, social functioning, role-emotional and mental health). Finally, at six weeks participants were more likely to believe a diagnosis of CRC was related to family history. There was also a decrease in their perceived risk of developing CRC in their lifetime following participation in CanPrevent, highlighting the importance of investigating beliefs regarding lifestyle factors and perceived risk of CRC.

With the limitations of a small acceptability and short-term effectiveness study in mind, the CanPrevent intervention results were very positive compared with the findings of previous investigators [20,21]. We observed improvements in multiple risk factors as well as HRQoL. It is also important to note that the improvements in HRQoL are considered clinically significant (2–5 point change) [61]. In comparison, PREVENT investigators observed significant intervention effects for multivitamin and red meat intake [21] and Bowel Health to Better Health had significant intervention effects on fibre intake alone [20]. The current study participants were also more likely to meet the national recommendations for most health behaviours post-intervention. These positive findings may be attributed to the fact that CanPrevent focused on multiple health behaviours which may have maximised the intervention effect. Further, CanPrevent was a novel ACT-based intervention that used strategies to overcome internal barriers to behavioural improvements by emphasizing the role of emotions and thoughts in the maintenance of good self-management of health behaviours. Consistent with the literature, it is possible that higher levels of physical activity in particular during participation in CanPrevent may have contributed to the observed improvements in HRQoL as physical activity has direct physical and mental health benefits [66]. Whilst the observed improvement in social functioning may have been a result of the regular telephone contact with the health coach during the intervention period. However, further research is required to confirm the positive findings of this short-term effectiveness trial, to identify mediators of the intervention effects, and to determine whether the observed improvements in health behaviours and HRQoL can be sustained.

There were a number of strengths to this study including: validated and reliable outcome measures that have been used over the telephone; a theory-based multiple behavior change intervention; a high rate of intervention delivery; a potentially low-cost, high-reach intervention; and a high level of interest and satisfaction with the intervention. Importantly, mediated (non-face-to-face) intervention delivery can be cost-effective and provide repeated contacts necessary to promote behavior change [67]. Telephone delivery is one of the most accessible mediated approaches and has potential for adoption by organisations that routinely operate telephone information and support centres [67]. The study was limited by use of self-report measures and their inherent biases, although all measures have been routinely used in population-based epidemiological and intervention research and over the telephone. Data were also collected by telephone interview which limited our ability to collect objective biomedical data. There were also more female than male participants, and the small sample size and lack of a control group were significant limitations. However this small pilot study was primarily designed to test the acceptability and short term effectiveness of the CanPrevent intervention.

## Conclusions

This study provides further support that comprehensive interventions focusing on a range of health behaviours can result in improvements in health outcomes. CanPrevent was acceptable and the results of this study suggest that the intervention may be effective in promoting multiple health behaviour, and HRQoL, improvements. A larger scale randomised controlled trial is required to confirm these findings and to determine longer term effectiveness.

## Abbreviations

CRC: Colorectal cancer; HRQoL: Health-related quality of life; MVPA: Moderate to vigorous physical activity.

## Competing interests

The authors declare that they have no competing interests.

## Authors’ contributions

ALH initiated the study and developed the study protocol. JFA and AG contributed to the final study protocol and study materials. TAP was responsible for implementing the study protocol. ALH drafted the manuscript and all authors contributed to the final version. All authors read and approved the final manuscript.

## Pre-publication history

The pre-publication history for this paper can be accessed here:

http://www.biomedcentral.com/1471-2407/12/560/prepub
